# Vomiting of Milk

**Published:** 1884-04

**Authors:** 


### VOMITING OF MILK.

IN GENERAL: Æthusa, Ant. crud., Arg. n., Arn., Borax, Bry., Calc., Calc. ph., Cham., Cina, Iod., Ipec., Lyc., Merc., Nux v., Rheum, Samb., Sil., Sulph.

CURDLED MILK: *Æthusa*, Ant. cr., Calc.

ÆTHUSA: Milk is forcibly ejected *soon* after it has been taken. The child is weak and *drowsy*. On awaking will nurse or feed again, only to vomit it soon after.

ANT. CRUD.: Child throws up some milk as soon as breast has been taken.

CHAM.: Milk vomited—is cheesy.

CINA: Babe refuses the milk. (Stannum.)

CALC. PH.: Babe refuses the breast. Milk tastes salty.

MERC.: Rejects milk.

RHEUM: Milk is yellow and bitter. Baby rejects the breast.

SILICEA: Aversion to milk. Refuses to nurse, and if does so, vomits.

